# Clinical associations of serum interleukin-17 in systemic lupus erythematosus

**DOI:** 10.1186/ar4277

**Published:** 2013-08-23

**Authors:** Fabien B Vincent, Melissa Northcott, Alberta Hoi, Fabienne Mackay, Eric F Morand

**Affiliations:** 1 Department of Immunology, Monash University, Central Clinical School, Alfred Medical Research and Education Precinct (AMREP), 89 Commercial Road, Melbourne, VIC, 3004, Australia; 2 Monash University Centre for Inflammatory Diseases, Southern Clinical School, Monash Medical Centre, 246 Clayton Road, Clayton, VIC, 3168, Australia

**Keywords:** Autoimmunity, B cell activating factor of the tumour necrosis factor (TNF) family (BAFF), interleukin-6, interleukin-17, macrophage migration inhibitory factor (MIF), systemic lupus erythematosus (SLE), T helper 17 (Th17)

## Abstract

**Introduction:**

Serum interleukin (IL)-17 concentrations have been reported to be increased in systemic lupus erythematosus (SLE), but associations with clinical characteristics are not well understood. We characterized clinical associations of serum IL-17 in SLE.

**Methods:**

We quantified IL-17 in serum samples from 98 SLE patients studied cross-sectionally, and in 246 samples from 75 of these patients followed longitudinally over two years. Disease activity was recorded using the SLE Disease Activity Index (SLEDAI)-2k. Serum IL-6, migration inhibitory factor (MIF), and B cell activating factor of the tumour necrosis factor family (BAFF) were also measured in these samples.

**Results:**

Serum IL-17 levels were significantly higher in SLE patients compared to healthy donors (*P *<0.0001). No correlation was observed between serum IL-17 and SLEDAI-2k, at baseline or during longitudinal follow-up. However, we observed that SLEDAI-2k was positively correlated with IL-17/IL-6 ratio. Serum IL-17 was significantly increased in SLE patients with central nervous system (CNS) disease (*P *= 0.0298). A strong correlation was observed between serum IL-17 and IL-6 (r = 0.62, *P *<0.0001), and this relationship was observed regardless of disease activity and persisted when integrating cytokine levels over the period observed (r = 0.66, *P *<0.0001). A strong correlation of serum IL-17 was also observed with serum BAFF (r = 0.64, *P *<0.0001), and MIF (r = 0.36, *P *= 0.0016).

**Conclusions:**

Serum IL-17 concentration correlates poorly with SLE disease activity but is significantly elevated in patients with CNS disease. IL-17/IL-6 ratio may be more useful than IL-17 or IL-6 alone to characterize Th17-driven disease, such as SLE. The association of other cytokines with serum IL-17 suggests that IL-17 may drive activation of diverse immune pathways in SLE.

## Introduction

Systemic lupus erythematosus (SLE) is a chronic idiopathic systemic autoimmune disorder characterized by multiple organ damage, particularly the joints, skin, brain and kidneys [1]. This condition is characterized by both innate and adaptive immune dysregulation (reviewed in Vincent *et al. *[2]), but the relative importance of cytokines operative in these systems is unknown. Interleukin (IL)-17 is the prototypic T helper 17 (Th17) cell pro-inflammatory cytokine. Since the identification of Th17 cells as a third subset of CD4+ T effector cells, [3,4] there has been much interest regarding the role of the Th17 axis in the pathogenesis of autoimmune disease. IL-17 has been associated with the pathogenesis of a range of autoimmune diseases, including rheumatoid arthritis (RA) [5], systemic sclerosis [6], multiple sclerosis (MS) [7], and SLE [8]. IL-17 is recognized to have a central role in lupus-prone mouse models [9,10] and therefore presumed to be involved in human SLE pathogenesis (reviewed in Nalbandian *et al. *[8]). Although not observed in all studies [6], serum IL-17 levels have been found to be elevated in SLE patients compared to controls [11-13]. Correlations between serum IL-17 levels and SLE disease activity and anti-double stranded DNA (anti-dsDNA) antibody levels have previously been reported in some studies [11,13]. However, research into the potential relationships between IL-17 and the clinical expression of SLE has been limited, and data from longitudinally followed cohorts is lacking. Cytokines with diverse effects on the immune system such as IL-6 [14-17], B cell activating factor of the tumour necrosis factor family (BAFF), and macrophage migration inhibitory factor (MIF) have each been shown to have some interplay with IL-17 [18,19], and to have potential roles in the pathogenesis or clinical expression of SLE [2,20]. However, it is not clear whether IL-17 regulates the expression of other cytokines in patients with SLE.

In this study, we aimed to examine the associations between serum IL-17 levels and disease expression in SLE in terms of disease activity and organ involvement. We also analysed the relationship between serum IL-17 levels and serum levels of IL-6, BAFF, and MIF. The results indicate that although serum IL-17 levels do not correlate with standard measures of disease activity, it is increased in patients with CNS disease, and is strongly associated with serum concentrations of IL-6, BAFF, and MIF. Finally, our findings suggest that IL-17/IL-6 ratio may be a novel biomarker for disease activity in SLE.

## Material and methods

### Patients and clinical assessments

Patients aged over 18 fulfilling the 1982 American College of Rheumatology (ACR) criteria [21] for the classification of SLE were recruited between May 2007 and June 2009 from the Lupus Clinic of Monash Medical Centre, Melbourne, Australia. Written informed consent was obtained from each individual. Disease-related damage was recorded at baseline using the Systemic Lupus International Collaborating Clinics (SLICC) SLE Damage Index (SDI) [22]. Other baseline variables collected included patient age, gender, ethnicity and disease duration. Disease activity was recorded at each clinic visit using the SLE Disease Activity Index-2000 (SLEDAI-2k) [23]. Patients were recorded as having renal or central nervous system (CNS) involvement if they had evidence of involvement in ACR renal or CNS criteria, or on any renal or CNS domains of the SLEDAI-2k or SLICC-SDI at any time during the study period. Active renal disease was defined as a non-zero score in any of the renal components of the SLEDAI-2k. Patient follow-up was as scheduled by the treating physician. Patients received standard-of-care therapy. The Human Research Ethics Committee, Southern Health, approved the study protocol and materials.

### Samples

Paired sera and SLEDAI-2k scores were collected from patients at routine medical visits. Sera were stored at -20°C until use. Measurements of C-reactive protein (CRP), erythrocyte sedimentation rate (ESR), creatinine and anti-dsDNA antibodies (Abs) titres (normal <7; Farr assay, Anti-dsDNA kit, Trinity Biotech, Wicklow, Ireland), were performed. Healthy control samples were obtains from the Australian Red Cross Blood Service.

### Serum cytokine quantification

Sandwich enzyme-linked immunosorbent assay (ELISA) was used to determine serum concentrations of IL-16, IL-17, MIF, and BAFF. ELISA was performed using R&D human IL-17 DuoSet ELISA development kit (R&D Systems, Minneapolis, MN, USA). Ninety-six-well ELISA plates (Immunoplates, Nunc, Roskilde, Denmark) were coated with monoclonal mouse anti-human IL-17 antibody. Plates were blocked with 1% bovine serum albumin in phosphate-buffered saline after overnight incubation at 4°C. After washing, samples and standards of recombinant human IL-17 were added to the plate and incubated overnight. Biotinylated goat anti-human IL-17 antibodies and streptavidin conjugated to horseradish peroxidase were used for detection, followed by 3,3',5,5'- tetramethylbenzidine (Sigma-Aldrich, Sydney, NSW, Australia) to develop colour. Plates were read at 450nm against standard curves for IL-17. The detection limit of the assay was 16 pg/mL.

The above ELISA protocol was similarly completed using the human IL-6 DuoSet ELISA development kit (R&D Systems, Minneapolis, MN, USA). Similarly, MIF concentrations were measured using mouse monoclonal anti-human MIF antibodies (R&D Systems, Minneapolis, MN, USA) for capture and goat biotinylated anti-human MIF antibodies (R&D Systems, Minneapolis, MN, USA) for detection. Recombinant human MIF was used as a standard (R&D Systems, Minneapolis, MN, USA). 1% bovine serum albumin in 5% sucrose-phosphate-buffered saline was used as a blocking agent. For BAFF, a similar ELISA protocol was performed, using purified mouse anti-human BAFF antibodies (BD Biosciences, San Jose, CA, USA) for coating the plates, and standards of recombinant human BAFF (R&D Systems, Minneapolis, MN, USA), and biotinylated goat anti-human BAFF antibodies (R&D Systems, Minneapolis, MN, USA). The detection limit of the assay was 1.5ng/ml. Each sample was tested in duplicate.

### Statistical analysis

Data are expressed as median and interquartile range (IQR), except where otherwise indicated. As serum cytokine concentrations were not normally distributed, nonparametric analysis was used in this study. Spearman's rank test was used to analyze correlations between variables. Mann-Whitney *U *tests and chi-square tests were used to compare groups of continuous and categorical data respectively. A *P *value of <0.05 was considered statistically significant. All tests were two-tailed unless otherwise stated. Statistical analyses were performed using GraphPad software (Prism Version 5.0D, 2010, San Diego, CA, USA).

## Results

### Patient characteristics

A total of 343 paired sera and SLEDAI-2k scores were collected from 98 SLE patients. The median (IQR) age and disease duration at baseline were 40 (31 to 52) years and 9 (4 to 15) years respectively. Thirty-nine (39.9%) patients were Asian and 57 (58.2%) were Caucasian. Baseline disease activity (*n *= 98) was highly variable, with SLEDAI-2k scores ranging from 0 to 26, with a median of 4. Seventy-five of these patients were studied longitudinally on a total of between two and ten occasions (median (IQR) number of visits = 3 (2 to 5); median (IQR) time between visits = 12 (7 to 21) weeks). Accordingly, time-adjusted means (TAM) were calculated for patients with more than one sample (*n *= 75). Serum anti-dsDNA antibody levels correlated positively with SLEDAI-2k (r = 0.43, *P *<0.0001) in this cohort, as did SLICC-SDI scores with time-adjusted mean SLEDAI-2k (r = 0.24, *P *= 0.022) and disease duration (r = 0.51, *P *<0.0001) (data not shown). Twenty-nine (29.9%) patients had CNS involvement. The majority (57.3%) of patients were receiving prednisone or equivalent, amongst whom the median (IQR) prednisone or equivalent dosage was 5 (0 to 10) mg/day.

### Associations between serum IL-17 and SLE disease activity

**Figure 1:**
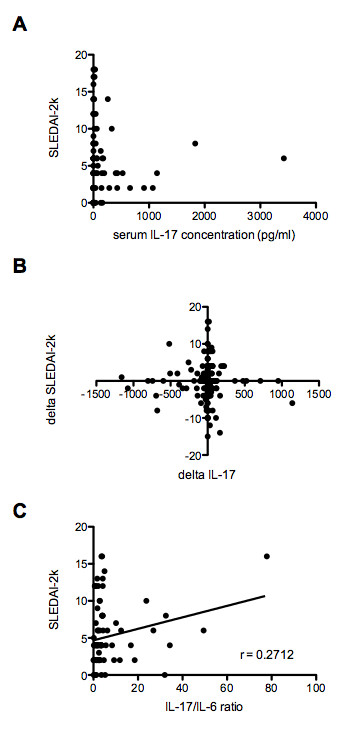
**Association of serum IL-17 concentrations with disease activity at a single point in time and over time**. **(A) **Correlation of serum IL-17 concentrations with SLEDAI-2k in SLE patients. No significant correlations were observed. **(B) **Between-visit changes in disease activity (delta SLEDAI-2k) was plotted against between-visit changes in serum IL-17 concentrations (*n *= 246). No significant correlations were observed. **(C) **SLEDAI-2k significantly correlated with IL-17/IL-6 ratio in all samples, after removing both undetectable IL-6 and IL-17. Serum IL-17 levels are expressed in pg/ml. IL-17, interleukin-17; SLE, systemic lupus erythematosus; SLEDAI-2k, SLE Disease Activity Index 2000.

We endeavoured to clarify the association of Th17 cytokine measurement with SLE disease activity, taking the advantage of cross-sectional and longitudinal follow-up of these patients. At baseline, serum IL-17 was undetectable in 39 patients, and concentrations were broadly distributed, ranging from 0 to 3423 pg/ml. Baseline median (IQR) serum IL-17 was 0 (0 to 63) pg/ml, and mean (standard deviation) serum IL-17 was 140.6 (434.9) pg/ml. Serum IL-17 was undetectable in 37 of 39 healthy donors and their median (IQR) serum IL-17 was 0 (0 to 0) pg/ml. Serum IL-17 levels were significantly higher in SLE patients compared to healthy donors (*P *<0.0001). In SLE patients, there were no differences in serum IL-17 concentration among SLE patients according to ethnicity or gender. No correlation was present between serum IL-17 levels and SLE disease activity assessed by SLEDAI-2k, at baseline (Figure 1A). There was no relationship between serum IL-17 and anti-dsDNA antibodies, CRP, ESR, or prednisone dosage, and there was no difference between patients using or not using glucocorticoids (data not shown). In samples separated categorically by disease activity, there were no difference in serum IL-17 concentrations between patients with inactive disease (SLEDAI-2k <4) and those with active disease (SLEDAI-2k ≥4) or those with higher levels of disease activity (SLEDAI-2k ≥8, 12, 16, or 18) (data not shown).

We next characterized relationships between change in disease activity and change in serum IL-17 levels in 246 samples from 75 patients followed up on at least one occasion after the first visit. In order to account for differences in baseline between patients, we compared changes in serum IL-17 levels (ΔIL-17) between consecutive visits with corresponding changes in disease variables. There were no correlations between ΔIL-17 and ΔSLEDAI-2k (Figure 1B), ΔESR, ΔCRP, Δanti-dsDNA Abs, Δcreatinine or Δprednisone dosage (data not shown). The adjusted mean SLEDAI (AMS) is a derivation of SLEDAI, calculated as the area under the curve divided by the time observed, which allows the comparison between patients of variation in SLEDAI integrated over time and has been shown to predict mortality [24,25]. TAM serum IL-17 levels were calculated for the 75 patients with multiple samples. There was no relationship between AMS and TAM IL-17.

### Associations between IL-17/IL-6 ratio and SLE disease activity

Cell subset ratios relevant to Th17 activity, such as Th17/regulatory T cells (Treg) ratio, Th17/Th1 cell ratio or Th1/Th2 cell ratios, have been reported to exhibit correlation with disease activity in autoimmune disease [26-29]. In the majority of such studies, *ex vivo *cytokine production ratios were used to represent cell populations [30]. Ratios of cytokine concentrations within the same axis may also be a marker of immune pathway activation, as has been reported for IL-17/TGF-β ratio in schizophrenia or IL-17F/IL-22 ratio in chronic hepatitis C virus infection [31,32]. We aimed to determine whether the ratio of IL-17 and IL-6, two major cytokines involved in the Th17 pathway, could provide a useful marker for disease activity in SLE. When expressed as a ratio of IL-17 to IL-6 in the same patient, SLEDAI-2k significantly correlated with IL-17/IL-6 ratio in all samples, after removing those where both IL-6 and IL-17 were undetectable (r = 0.2712, *P *= 0.0131) (Figure 1C).

### Serum IL-17 association with the presence of CNS disease in SLE

**Figure 2:**
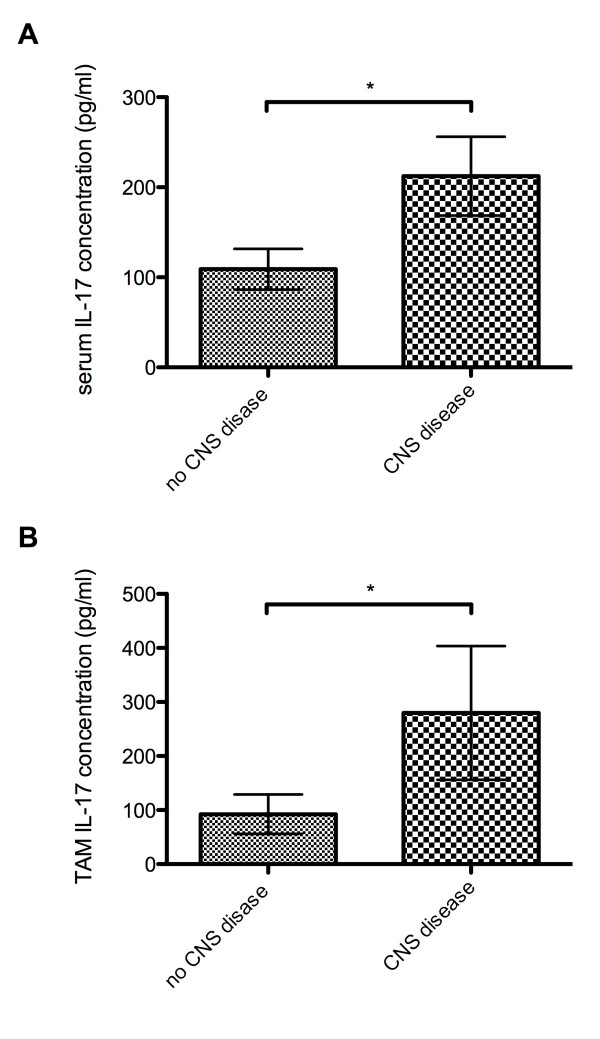
**Association of serum IL-17 concentrations with the presence of CNS disease in SLE patients**. **(A) **Serum IL-17 concentrations in SLE patients with and without CNS involvement. *P <0.05. **(B) **TAM serum IL-17 concentrations in SLE patients with and without CNS involvement. *P <0.05. Serum IL-17 levels are expressed in pg/ml. CNS, central nervous system; IL-17, interleukin-17; SLE, systemic lupus erythematosus; TAM, time-adjusted mean.

We also aimed to characterize the association of Th17 cytokine measurement with SLE expression in terms of organ involvement. Serum IL-17 concentrations were significantly increased in SLE patients with a history of CNS disease (*P *= 0.0298) (Figure 2A). Similarly, TAM IL-17 levels were increased in patients with CNS disease (*P *= 0.0495, one-tailed) (Figure 2B), and correspondingly patients with TAM IL-17 levels above the 75^th ^percentile had a higher prevalence of CNS disease compared to the remaining patients (relative risk (RR) = 2.0, 95% confidence interval (CI) 1.1 to 3.7). In contrast, no change in serum IL-17 levels was observed regardless of the presence or absence of renal disease at baseline or in longitudinal follow-up (data not shown). Of note, however, serum IL-17 concentration negatively correlated with creatinine levels (r = -0.2836, *P *<0.0001) (data not shown). In the subgroup of SLE patients with CNS disease, serum IL-17 levels negatively correlated with prednisone dosage (r = -0.247, *P *= 0.0066) (data not shown). In addition, in the subgroup of CNS SLE patients, both of serum IL-6 and IL-17 levels were correlated with ESR (r = -0.208, *P *= 0.0235, and r = -0.2501, *P *= 0.006, respectively) (data not shown).

### Correlation of serum IL-17 with other cytokines

**Figure 3:**
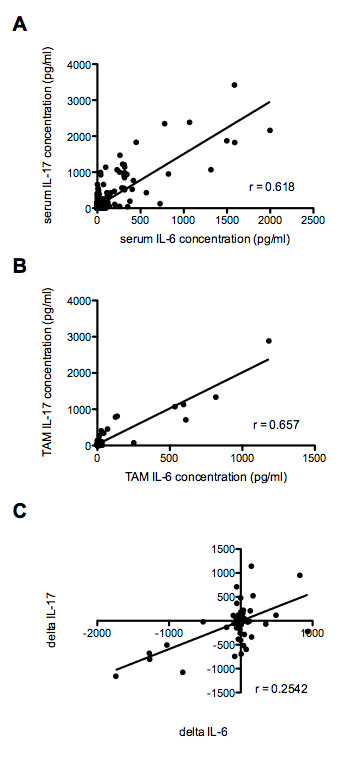
**Correlation of serum IL-17 concentrations with serum IL-6 concentrations in SLE patients**. **(A) **Correlation between serum IL-6 and serum IL-17 levels in all samples. There was a strong positive correlation (r = 0.618, *P *<0.0001). **(B) **Correlation between TAM serum IL-6 and TAM serum IL-17 levels. There was a strong positive correlation (r = 0.657, *P *<0.0001). **(C) **Correlation between changes in serum IL-6 (ΔIL-6) and IL-17 (ΔIL-17) levels between visits. There was a strong positive correlation between ΔIL-6 and ΔIL-17 (r = 0.2542, *P *<0.0001). Serum IL-6 and IL-17 levels are expressed in pg/ml. IL, interleukin; SLE, systemic lupus erythematosus; TAM, time-adjusted mean.

Because of the potential of IL-17 to regulate other pro-inflammatory cytokines, we analysed correlations between serum IL-17 concentration and those of three other cytokines associated with SLE. A strong positive correlation was present between serum IL-6 and IL-17 levels both in the 98 baseline samples and when all samples were considered (r = 0.6508, *P *<0.0001; and r = 0.618, *P *<0.0001, respectively) (Figure 3A). This correlation remained significant throughout all analysed subsets of samples, including when samples with undetectable IL-17 were excluded (r = 0.62, *P *<0.0001). The association appeared independent of disease activity, as it was similar in the subset of samples from patients with active disease (SLEDAI-2k >4; r = 0.49, *P *<0.0001), and with active renal disease (renal SLEDAI-2k >0; r = 0.50, *P *<0.0001). This relationship also persisted over longitudinal follow-up. TAM serum IL-6 levels correlated with TAM serum IL-17 levels (r = 0.657, *P *<0.0001) (Figure 3B). We also measured changes in serum IL-6 (ΔIL-6) and IL-17 (ΔIL-17) levels between visits. There was a strong positive correlation between ΔIL-6 and ΔIL-17 (r = 0.2542, *P *<0.0001) (Figure 3C).

**Figure 4:**
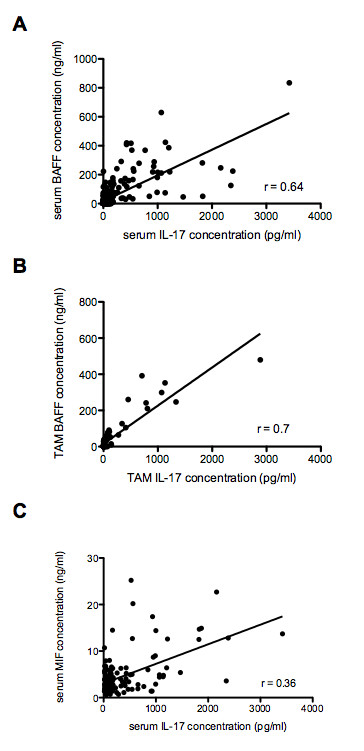
**Correlation of serum IL-17 concentrations with both serum BAFF and MIF concentrations in SLE patients**. **(A) **Correlation between serum IL-17 and serum BAFF levels in all samples. A strong positive correlation was present (r = 0.64, *P *<0.0001). **(B) **Correlation between TAM serum IL-17 levels and TAM serum BAFF in all samples. A strong positive correlation was present (r = 0.70, *P *<0.0001). **(C) **Correlation between serum IL-17 and serum MIF (after removing undetectable IL-17 levels). A strong positive correlation was present (*n *= 132, r = 0.36, *P *<0.0001). Serum IL-17 levels are expressed in pg/ml. Serum BAFF and MIF levels are expressed in ng/ml. BAFF, B cell activating factor of the tumour necrosis factor family; IL, interleukin; MIF, macrophage migration inhibitory factor; SLE, systemic lupus erythematosus; TAM, time-adjusted mean.

A strong positive correlation was also observed between serum IL-17 and serum BAFF levels in all samples (r = 0.64, *P *<0.0001) (Figure 4A). This remained consistent through all analysed subsets of samples, including samples with detectable levels of BAFF (r = 0.73, *P *<0.0001), samples from patients with active disease (SLEDAI-2k >4, r = 0.63, *P *<0.0001), and with active renal disease (renal SLEDAI-2k >0, r = 0.75, *P *<0.0001). TAM serum BAFF levels were also strongly correlated with TAM serum IL-17 (r = 0.70, *P *<0.0001) (Figure 4B). Whilst no significant correlation was present between serum MIF and serum IL-17 when all samples were analysed (data not shown), a positive trend was present, and a positive correlation emerged when samples without detectable levels of IL-17 were removed from the analysis (*n *= 132, r = 0.36, *P *<0.0001) (Figure 4C).

## Discussion

The role of IL-17 in the pathogenesis of autoimmune diseases is now well accepted, and as such, IL-17 is a potential therapeutic target. In this study, we report that serum IL-17 levels were significantly elevated in the serum of SLE patients compared to healthy controls, and among SLE patients with CNS involvement compared to those without. Moreover, we observed that IL-17/IL-6 ratio significantly positively correlated with disease activity assessed by SLEDAI-2k. We also report strong correlations between serum levels of IL-17 and serum IL-6 and BAFF, as well as a weaker, yet significant, correlation between serum IL-17 and serum MIF. These results suggest that these three cytokines may all potentially interact with the Th17-mediated pathways and may be involved with expression of IL-17-mediated inflammation or conversely with the regulation of IL-17 production.

We found no correlation between SLEDAI-2k score and serum levels of IL-17, IL-6, or MIF, as well as between ΔSLEDAI-2k and changes in these cytokines levels, in any subset of our samples. Previous reports of these relationships have been inconsistent. One publication reported a correlation between SLEDAI and serum IL-17 [13], whilst another reported a correlation in only patients without renal disease [11]. Conversely, other studies showed no correlation in any patient subset [33,34]. The reason for these discrepancies is unknown, but may be partially attributable to the relatively small sample sizes used in some studies. ELISA sensitivity as well as uncontrolled confounding factors, such as immunosuppressive medications, may influence serum cytokine levels. Of note, SLE is a highly heterogeneous disease, and it is likely that disease expression may differ between different patient groups, especially regarding major organ involvement, which may have impacted this result. In addition, serum IL-17 levels may not be an accurate reflection of total IL-17 production. IL-17 may be localized to the inflamed tissue in some cases, such as the brain, and therefore serum IL-17 levels may not be a faithful reflection of its endogenous production. However, we observed that SLEDAI-2k was significantly positively correlated with IL-17/IL-6 ratio. Thus IL-17/IL-6 ratio may more accurately characterize Th17-driven disease than serum IL-17 or IL-6 alone. To the best of our knowledge, this is the first published data that reported correlation between SLE disease activity and such a cytokine ratio.

As SLE is a highly heterogeneous disease, characterizing subgroups of SLE patients with specific disease phenotypes, such as major organ involvement, may improve the investigation of SLE pathogenesis. Considering SLE in terms of phenotypic subgroups may permit identification of patients with pathogenic pathways that may be amenable to targeted therapy. Cytokine measurement may help to characterize these pathways, and to identify potential therapeutic targets. In the current study, serum IL-17 levels were significantly elevated in SLE patients with CNS involvement. Our results suggest IL-17 is involved in CNS SLE pathogenesis, and represents a possible biomarker of CNS SLE and therapeutic target in this subgroup. In this study, the percentage of individuals affected with CNS SLE was higher than in previously reported cohorts. Asian SLE patients had significantly more history of CNS disease than Caucasian SLE patients (*P *<0.05), and the high representation of Asian ethnicity in this cohort may explain this difference. It would also be of interest to examine relationships between serum IL-17 levels and CNS involvement in relation to anti-phospholipid antibody status in future studies. Recent data suggest that Th1 cells act to facilitate the entry of Th17 cells in the CNS, which mediates the subsequent release of chemokines and recruitment of other inflammatory cells [35]. Both IL-17 and IL-6 are highly expressed in MS lesions [7], and IL-17 is elevated in the serum and cerebrospinal fluid (CSF) of these patients [36]. An *in vitro *study has shown that human Th17 cells may be well equipped to breach the blood-brain barrier and infiltrate the CNS parenchyma [37]. In addition, Cua *et al*. showed that IL-23p19^-/- ^mice characterized by an IL-17 deficiency, were resistant to experimental autoimmune brain inflammation, suggesting IL-23 (and thereby IL-17) as a crucial cytokine in autoimmune CNS diseases [38]. Meyers *et al*. showed that type I interferon (IFN) can inhibit IL-17 production in human peripheral blood mononuclear cells (PBMCs), suggesting a repressive effect of type I IFN on Th17 cells [39]. Studies investigating the relationship between IL-17 and type I IFN in human SLE would be of interest. Of note, IFN-gamma is also known to have a suppressive effect on Th17 development (reviewed in Shah *et al. *[40]). Shah *et al*. suggested that IL-17 production by Th17 cells in SLE is the consequence of dysregulation of Th17/Th1 balance [40]. Interestingly, Prado *et al*. recently showed that Th17/Th1 ratio was increased in SLE patients using glucocorticoids, compared to healthy controls [27]. Such an effect was not supported by our finding of equivalent serum IL-17 regardless of corticosteroid use.

Immunosuppressant drugs used in SLE could also impact on serum IL-17 concentrations, and thereby may be a confounding factor when analysing correlation between serum cytokines and disease activity. In our study, there was no relationship between serum IL-17 and prednisone dosage, and there was no difference between patients using or not using glucocorticoids. Assessment of immunosuppressant drug effects on serum IL-17 would be of interest; however, as we analysed patients followed for up to 10 serial visits, many changes in drug therapy occurred in individual cases, and together with variation in corticosteroid dose and disease activity the data do not allow us to make useful analyses of this question.

Clinical trials involving biologics targeting IL-17 are currently under way in several immune diseases. Secukinumab (AIN457) is a fully human anti-IL-17A IgG1κ monoclonal antibody (mAb), being studied in clinical trials in psoriasis, RA, Crohn's disease (CD), psoriatic arthritis (PsA), ankylosing spondylitis and MS. Although secukinumab was not effective in CD [41] and did not meet the primary endpoint in a phase II clinical trial in RA [42], it appears to be a promising therapy in phase II clinical trials in psoriasis [43]. Ixekizumab (LY2439821), a humanized IgG4 anti-IL17A mAb has showed efficacy in phases I and II in RA [44] and psoriasis [45], respectively, and is currently being studied in RA, PsA and psoriasis. Brodalumab (AMG 827), an anti-IL-17R mAb, is also currently being studied in clinical trials in RA, PsA, psoriasis, CD and asthma, with clinical improvement in psoriasis in phase II [46]. At present, no clinical trials of anti-IL-17 therapy are under way in SLE. The current findings suggest that CNS disease may be a particular target group for anti-IL-17 therapy in SLE.

Although not previously reported, the strong correlation found between serum IL-6 and IL-17 levels was in line with expectations, as IL-6 is one of the inflammatory cytokines required, in combination with TGF-β, for naïve T cells to differentiate into Th17 cells (reviewed in Nalbandian *et al. *[8]). Prospective analysis of changes in serum IL-6 and IL-17 concentrations in over 300 samples is a strength of this study, and we found that changes in serum IL-6 and IL-17 levels between visits were correlated, as were TAM IL-6 and TAM IL-17. Other cytokines such as IL-1β, TNF-α, IL-21, and IL-23 are also essential for sustaining IL-17 production. Furthermore, IL-17 has been shown to upregulate IL-6 production in mice, suggesting that a positive feedback loop may operate between the Th17 cell pathway and IL-6 [47]. Dong *et al*. recently showed that IL-17 can increase IL-6 in PBMCs from lupus nephritis patients [48].

The observed relationship between MIF and IL-17 is also interesting. MIF is an important cytokine in a variety of autoimmune diseases and in particular SLE. MIF is a known activator and facilitator of extracellular signal-regulated protein kinase (ERK), and recent reports suggest extracellular signal-regulated kinase (ERK) mitogen-activated protein kinase (MAPK) is involved in IL-17 production [19,49]. It has recently been reported that IL-17 as well as IL-6 and IL-23 production was downregulated in the lymph nodes of MIF-deficient mice, suggesting MIF plays a role in Th17 differentiation and IL-17 production [50]. Whether MIF could also induce and/or enhance IL-17 production has not been reported in a human disease state. To our knowledge, this is the first report suggesting such a relationship in SLE.

We observed that serum BAFF levels correlated with IL-17, as well as TAM BAFF with TAM IL-17, suggesting a relationship between the BAFF system and Th17 cells in human SLE. Indeed, one of the BAFF receptors, BAFF-receptor (BAFF-R or BR3) is expressed on activated T cells and Tregs (reviewed in Vincent *et al. *[2]). Dong *et al*. has shown that IL-17 increased IL-6 and anti-dsDNA antibodies in supernatant of culture PBMCs from lupus nephritis patients [48], suggesting a role for IL-17 in human B cell activation [8]. Supporting this, Hsu *et al*., showed that autoimmune BXD2 mice, which spontaneously produce high IL-17 levels, develop less germinal centre B cells as well as reduced humoral responses when IL-17R is lacking [9]. In a recent study, it has been demonstrated that BAFF can affect Th17 cell response in mice [51]. Using BAFF transgenic (BAFF-Tg) and BAFF knockout (KO) mice, it was demonstrated that BAFF drives Th17 cells induction and increased IL-6R expression. Of note, CNS infiltration of Th17 cells was reported to be higher in BAFF-Tg mice, and lower in BAFF KO mice, with autoimmune brain inflammation. In healthy humans, a correlation was reported between circulating Th17 frequency and BAFF levels as well as switched-memory B cells [52]. As, in turn, IL-17 is associated with IL-6 expression, BAFF may be a key inducer and stimulator of the IL-6/IL-17 loop in human SLE.

## Conclusions

In summary, in our study SLE patients with CNS disease had significantly upregulated IL-17 levels compared to patients without CNS involvement, suggesting IL-17 as a potential biomarker and therapeutic target in CNS SLE. Our results demonstrate for the first time in SLE a human correlate of previously published murine studies demonstrating the relationships of IL-6, BAFF and MIF with IL-17 production. These data increase support for investigation of IL-17 as a therapeutic target in SLE. Measurement of IL-17 in patients treated with anti-BAFF monoclonal antibodies could illuminate these relationships further.

## Abbreviations

Abs: antibodies; ACR: American College of Rheumatology; AMS: adjusted mean SLEDAI; dsDNA: double-stranded DNA; BAFF: B cell activating factor of the tumour necrosis factor family; BAFF-R: BAFF-receptor; BAFF-Tg: BAFF transgenic; CD: Crohn's disease; CNS: central nervous system; CRP: C-reactive protein; CSF: cerebrospinal fluid; ELISA: enzyme-linked immunosorbent assay; ERK: extracellular signal-regulated kinase; ESR: erythrocyte sedimentation rate; HD: healthy donors; IFN: interferon; IL: interleukin; IQR: interquartile range; KO: knockout; mAb: monoclonal antibody; MAPK: mitogen-activated protein kinase; MIF: migration inhibitory factor; MS: multiple sclerosis; PBMCs: peripheral blood mononuclear cells; PsA: psoriatic arthritis; RA: rheumatoid arthritis; SDI: SLE Damage Index; SLE: systemic lupus erythematosus; SLEDAI: SLE Disease Activity Index; SLICC: Systemic Lupus International Collaborating Clinics; Th17: T helper 17; TAM: time-adjusted means; Treg: regulatory T cells.

## Competing interests

The authors declare that they have no competing interests.

With the financial support of the French Society of Rheumatology and Arthritis, Victoria.

## Authors' contributions

EM designed data collection tools, and monitored data collection for the whole study. FV, MN, AH, FM and EM contributed to the study conception and design, and data interpretation. FV and MN contributed to data acquisition. FV and EM wrote the statistical analysis plan, collected, cleaned and analysed the data, and drafted the manuscript. FV, MN, AH, FM and EM analysed the data, read and revised the draft manuscript. All authors read and gave final approval of the version to be published.
